# Effects of Heat-Treatment on Tensile Behavior and Dimension Stability of 3D Printed Carbon Fiber Reinforced Composites

**DOI:** 10.3390/polym13244305

**Published:** 2021-12-09

**Authors:** Amal Nassar, Mona Younis, Mohamed Elzareef, Eman Nassar

**Affiliations:** 1 Mechanical Engineering Department, Higher Technological Institute, Next to Small Industries Complex, Industrial Area 2, 10th of Ramadan City 11111, Egypt; Mona.Younis@hti.edu.eg (M.Y.); eman.nasser@hti.edu.eg (E.N.); 2 Mechanical Engineering Department, The British University in Egypt, Suez Desert Road, El Sherouk City 11837, Egypt; mohamed.elzareef@bue.edu.eg

**Keywords:** heat treatment, 3D printed, fiber-reinforced composites and tensile behavior

## Abstract

This work investigated the effects of heat treatment on the tensile behavior of 3D-printed high modules carbon fiber-reinforced composites. The manufacturing of samples with different material combinations using polylactic acid (PLA) reinforced with 9% carbon fiber (PLACF), acrylonitrile butadiene styrene (ABS) reinforced with 9% carbon fiber (ABSCF) were made. This paper addresses the tensile behavior of different structured arrangements at different% of densities between two kinds of filaments. The comparison of the tensile behavior between heat treated and untreated samples. The results showed that heat treatment improves the tensile properties of samples by enhancing the bonding of filament layers and by reducing the porosity content. At all structure specifications, the rectilinear pattern gives higher strength of up to 33% compared with the Archimedean chords pattern. Moreover, there is a limited improvement in the tensile strength and modulus of elasticity values for the samples treated at low heat-treatment temperature. The suggested methodology to evaluate the tensile behavior of the pairs of materials selected is innovative and could be used to examine sandwich designs as an alternative to producing multi-material components using inexpensive materials.

## 1. Introduction

Additive manufacturing (AM) is a new production technique, commonly called 3D printing, used for metal and polymer processing [1,2,3]. Products manufactured using AM are designed by adding material layer by layer, while products are built using subtractive manufacturing processes in traditional methods [4,5,6]. The cheap cost and adaptability in the construction of complicated designs have expanded the use of AM in current applications [7], such as development of products in the automotive industry, aerospace/biomedical applications, arts and design development, architecture, and so on [1,2,8,9]. Due to its growing use in the above stated applications, AM’s development has grown strongly after 2013 [10]. Currently, aeronautical, electrical, and medicinal applications [11] are often employed in AM technology [12,13,14]. In addition to that, Tadesse et al. [15] use the three-dimensional (3D) printing to develop a flexible and lightweight electroluminescence (EL) device as a cost-effective technique for EL applications. AM is a flexible production technique that develops the product directly from the design file, decreasing the lead time for the product and material waste, and economically creates a complicated design [16]. Recently, many investments have been carried out to produce aircraft components by using AM, including brackets, panels, clips, and supports. Airbus used about 2700 components in A350 WXB aircraft made by using AM technology. Furthermore, South African company, Aerosud, has ordered more than 80 polymer components produced by AM technology for its aircraft to be used as air guidance ducts and low load-bearing air intakes. Using AM technology has reduced the cost and overall weight compared to using conventional metal parts [17].

However, there are a few problems in the AM process, such as, slow mass production and limited material use, which restrict it in several applications. AM has very selective material applications [18,19,20], since the additive manufactured materials are largely found only as a prototype model [21]. In this regard, the multiple printing head technique has been developed where composite materials can be created using controlled material combinations and properties [12]. The development of fiber composites is a more challenging approach for the AM process. Several aspects need to be considered for the development of fiber composites, including fiber weight percentage. Because of their excellent performance, manufacturing facility, and low cost, polymers are the materials most frequently utilized in various applications. In AM polymers, such reactive, fluid- and thermoplastic melts, are used in a range of different forms [22].

Industry has recently focused on the development of multilayered polymer materials and sandwich structure techniques for the Fused Deposition Modeling (FDM) process. Multilayer materials can improve the quality of 3D printing by optimizing the structure of various processes, allowing smart components to work [16,18]. Multi-material component manufacturing effectively increases mechanical properties, allows for new features, and improves AM process performance [23]. Furthermore, sandwich structure made from a mixture of polymer combinations, such as lightweight interior components for automobiles, has long been believed to be a suitable way to obtain a diversity of material properties for customized items. The outer skin of composite materials made of high-strength material sandwich the inner core of a lightweight material, which is covered with sandwich structures [21]. The inner core is usually made up of a wave structure because of its weight. However, problems, such as water ingress and delamination, may occur [24]. The necessary strength and stiffness would be attained by the difference between the skin material and the core, according to Daniel and Abot [25]. The optimum design for creating a lightweight composite appears to be more exact in the core material than in the thickness of the core layer, according to the findings of Herranen et al. [26]. For homogenous FDM thermoplastics, Lanzotti et al. examined the influence of process variables such as layer thickness, flow rate, deposition speed, feed rate, and build orientation on single PLA specimens [22,27]. Initially, it was found that all fibers must go down the loading line to maximize their Young’s modulus and stiffness. On-line samples showed the best performance in terms of strength, stiffness, and ductility. Furthermore, increasing the layer’s thickness and feeding rate reduces its ductility. Kuznetsov et al. [28] examined basic thickness and PLA infill density to enhance the form of FDM 3D-printed material, which can withstand larger stresses. The inclusion of certain volume features such as the fillet, roundness, and smooth contours to these process parameters has been found to enhance component strength more than twice that required to break the identical component while also reducing component mass considerably [29]. Fernandez-Vicente et al. [30] investigated the strength of different mesostructured ABS generated by the FDM technique to determine the optimum specimen, a tensile test was performed, which comprised material densities and infilling patterns. The best combination for greatest tensile strength was a rectilinear design with 100 percent infill density. To discover the best combination of ABS, PLA, and High impact polystyrene HIPS, researchers looked at a variety of materials. Mechanical testing was carried out by Singh et al. and Kumar et al. [31,32]. The three materials were printed using the twin extrusion (TSE) technique as a stack of different multi-layers in the same shape. Saad [33] created an ABS and PLA sandwich structure to test the diversity of their mechanical and physical properties. Various percentages of honeycomb pumps were used in the study to test and validate the weight benefit of pore size volumes on mechanical properties. The results of tensile and bending tests showed that if the infill density increases while the steadiness remains constant as the bending force increases, the tensile strength increases. Furthermore, 3- and 4-point ABS and PLA sandwich bending tests were recommended by Brischetto et al. [34]. Infill patterns such as the core layers (honeycomb and homogenous) and the number of extractors employed to make specimens have an impact. The modulus of elasticity of ABS and PLA honeycomb core, in particular, was impacted by all of these variables. In order to improve their mechanical performance, Santosh et al. [35] utilized layered structures using ABS and PLA. 

Heat treatment of polymer materials made by perfecting the interfacial bonding to improve the mechanical properties [36,37]. However, the studies of the effect of heat-treatment on 3D-printed composites remain limited. MacDonald et al. [38] found that porosity and mechanical properties are affected by heat treatment of the polyether ether ketone produced by ASTM 52900 process. Nabipour et al. [39] studied the relationship between annealing and increasing tensile strength of (PLA) 3D-printed samples. They noticed that heat-treatment can improve the interface bonding which leads to an increase in the density. Wang et al. suggested that the heat treatment process can improve the tensile strength of poly wax 3D-printed samples by reducing the amount of porosity. Some researchers studied the tensile behavior of 3D-printed PLA reinforced by short carbon fiber SCF after heat treatment process [40]. Natalia et al. [41] studied the influence of porosity, crystallinity, and interlayer adhesion on the tensile strength of 3D-printed polylactic acid (PLA). Their final results prove that medical diameter and layer height are the most significant factors that affect the mechanical properties of 3D-printed parts. Similar results were postulated by Travieso-Rodriguez et al. [42]. Afonso [43] et al. studied the influences of the fused filament fabrication parameters process on the mechanical properties for printed polylactic acid (PLA) parts. They found that the greatest influence parameter in the fabrication process is the extrusion temperature. Gardner et al. [44] proved that the annealing process enhanced the tensile properties of 3D-printed composites reinforced with SCF. Thus, it can be concluded that an annealing process can affect the porosity, interface bonding, and crystallinity of polymer for 3D-printed composites. Nevertheless, the synergistic effects of the annealing process on the mechanical behavior and microstructure require more study. Although, as per the authors’ knowledge, no research has focused on the relevance of heat-treatment, matrix type, and infill density and pattern. This work investigates the effect of using heat treatment as a solution to the poor mechanical properties of 3D-printing products. The novel structure of the sandwich is suggested here, with specimens incorporating rectilinear cores and Archimedes with an infill density of 100 percent and 70 percent for each material inside a single carriage and employing many independent nozzles extruders. In this novel scheme, the effect of combining traditional (ABSCF and PLACF) materials as a sandwich structure for achieving and improving the greater strength of polymer elements that may be employed in diverse applications, could be compared.

## 2. Experimental Work

### 2.1. Materials

The test materials are acrylonitrile butadiene styrene with 9% concentration of carbon fibers (ABSCF) (CarbonX™, 3DXTECH, Grand Rapids, MI, USA) and polylactide with 9%concentration of carbon fibers (PLACF) (CarbonX™, 3DXTECH, Grand Rapids, MI, USA). The properties of both filaments according to the 3dxtech data sheet are shown in Table 1 [45,46]. In FDM, the polymers are extruded and mounted in a product development layer by layer technique. FDM produced polymers show adequate mechanical performance, high surface quality, and reliability at reasonable prices compared to other AM techniques. The FDM method material is in the form of long wires or filament wound on as pool.

**Figure 1:**
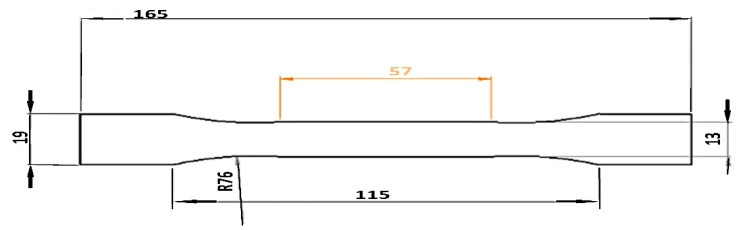
Dimension details of the tensile test sample according to ASTM D638.

Firstly, specimens were printed as a single homogeneous material with fixed parameters to be compared with the multi-material specimens. Secondly, the combination of materials for the sandwich structure was performed with four-layer sections of each material. It contained symmetrical 1.2-mm-thick polymeric inner and outer cores (four layers of 0.3 mm thick per material) giving a total dimension of a 3.6 mm thick specimen in accordance with the dimensions of the standard test method for tensile properties of plastics as shown in Figure 1. All specimens were printed in a flat direction on the XY plane with a rectilinear pattern, stacking sequence of 45°/45°, and vertically upward layer by layer within the Z direction. The air gap was recognized as zero (beads just touch). Sample configurations are:

- Type (A) for ABSCF: ABSCF 100/rectilinear; ABSCF 70/rectilinear; ABSCF 100/Archimedean chords; ABS 70/Archimedean chords,
- Type (A) for PLACF: PLACF 100/rectilinear; PLACF 70/rectilinear; PLACF 100/Archimedean chords; PLA 70/Archimedean chords,
- Type (B) for PLACF-ABSCF-PLACF: PLACF-ABSCF-PLACF 100/rectilinear; PLA-ABSCF-PLACF 70/rectilinear; PLACF-ABSCF-PLACF 100/Archimedean chords; PLACF-ABSCF-PLACF 70/Archimedean chords,
- Type (B) for ABSCF-PLACF-ABSCF: ABSCF-PLACF-ABSCF 100/rectilinear; ABSCF-PLACF-ABSCF 70/rectilinear; ABCFS-PLACF-ABSCF 100/Archimedean chords; ABSCF-PLACF-ABSCF 70/Archimedean chords,
- Type (B) for PLACF-ABSCF-PLACF: PLACF-ABSCF-PLACF 100/rectilinear; PLACF-ABSCF-PLACF 70/rectilinear; PLACF-ABSCF-PLACF 100/Archimedean chords; PLACF-ABSCF-PLACF 70/Archimedean chords.

The samples were divided into two groups. The first group was subjected to heat treatment and the other group was examined without any treatment.

### 2.2. Printing Process

**Figure 2:**
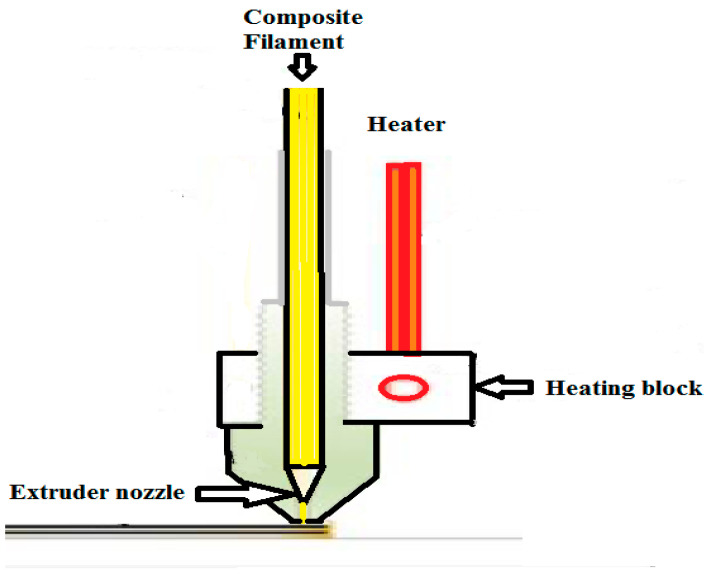
Schematic of using single head in manufacturing process.

**Figure A1:**
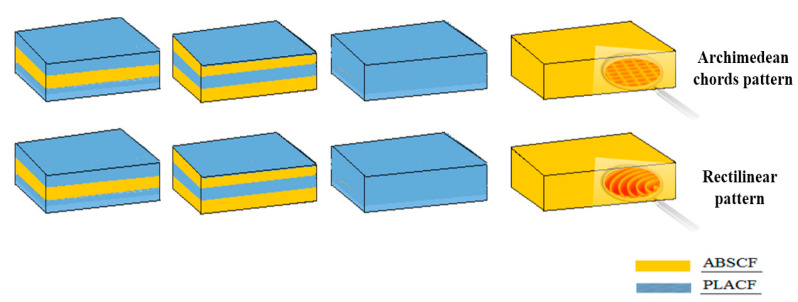
Layer distribution and infill patterns of the printed specimens.

Printing process of the selected type of composite filaments made by using a single head. In the printing process, multiple layers are used to produce the required sample (Figure 2). The FDM printing used is done by a 3D printer of type original Prusa MK3S (Prusa company, Prague, Czech Republic), according to layer distributions and infill patterns in Figure A1. The method proposes a sandwich structure with a total thickness of 3.6 mm, The two outer skins have a global thickness of 2.4 mm and the inner core a thickness of 1.2 mm made of a PLACF or ABSCF with a rectilinear or Archimedean chords infill pattern and 100% or 70% density, the speed rate was in accordance with the loading rate used in previous studies [22,27,29,30]. Table 2 shows parameters of the printing for each filament type. Table 3 illustrates the given code for each sample.

### 2.3. Heat Treatment

**Figure A2:**
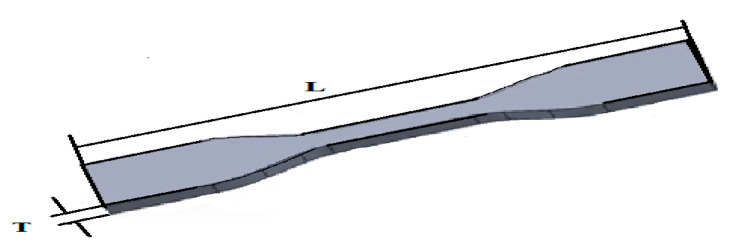
Locations to measure the dimensions of the sample.

Annealing or heat-treatment experiments were established to analyze the deformation caused by the annealing process in the dimensions length (L) and thickness (T) of the specimen as shown in Figure A2.

**Figure A3:**
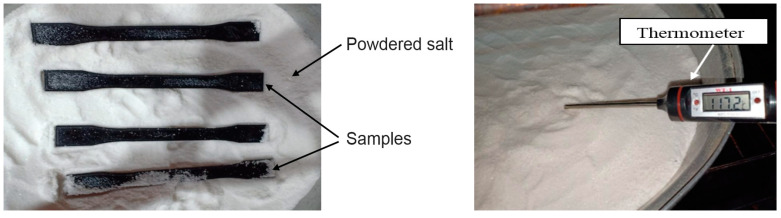
Annealing process.

The annealing process is done by first putting the specimens in a ceramic container and leaving around two inches of space around it on all sides. The powder used in the annealing process is a mixture of sodium chloride powder (table salt), potassium iodate 50 mg/kg, and E536 anti-caking agent. These are non-toxic, inexpensive materials, and high-temperature resistant, used to distribute the heat uniformly around the specimen and prevent deformation of the parts. The powders were mixed and ground into small particles (about 50 µm) and then heated to 200 °C for 40 min to remove moisture, then it was left to cool down in a sealed container. The resulting powder is added to the bottom of the container and then the specimens are placed on top of it as shown in Figure A3, then the sodium chloride powder is gently poured all around them. The electrical heater oven is pre-heated and left until it holds temperature for 10 min and then the container is inserted inside the oven and left for 20 min to make sure the salt has enough time to fully heat up and transfer that heat to the specimens.

A thermometer is inserted into the salt to make sure the internal temperature is the same as what the oven thermometer reads. The container with samples is left inside the oven for 10 min at the set temperature. After that, the container is left to cool down at room temperature (25 °C). This process is done at three different temperatures which are 50 °C, 120 °C, and 150 °C for all the sandwich specimens; these values are set in order to be above the glass transition temperature and below the melting point of the material used in the specimens, so for the sandwich specimens, an average value of the two materials is taken. Specimens’ thicknesses and widths, before and after annealing, were measured using a digital Vernier caliper to analyze the deformation that happened.

### 2.4. Characterization

The effect of heat-treatment over type A and type B samples with different kinds of filaments were studied. This study includes physical experiments and mechanical tests for the samples. The physical experiments include porosity and dimension measurements, and mechanical tests include tensile tests. The changes in the dimension under the effect of heat treatment was measured by using a digital Vernier caliper and the results were the average of 3 readings. The density measurement was used to calculate the porosity % by using Formula (1).

Porosity % = ({D (filament type) − D (Printed sample)}/D (filament)) × 100(1)

In this formula, the densimeter type (DH-300K DahoMeter digital electronic, Dongguan Hong Tuo Instrument Co., Dongguan, China) based on the standard D 792–07 was used to measure the density of filament type (ABSCF, PLACF) and the density of the printed sample; the value of each sample was recorded as an average of three readings.

## 3. Results and Discussion

### 3.1. Stability of the Dimension

**Figure 3:**
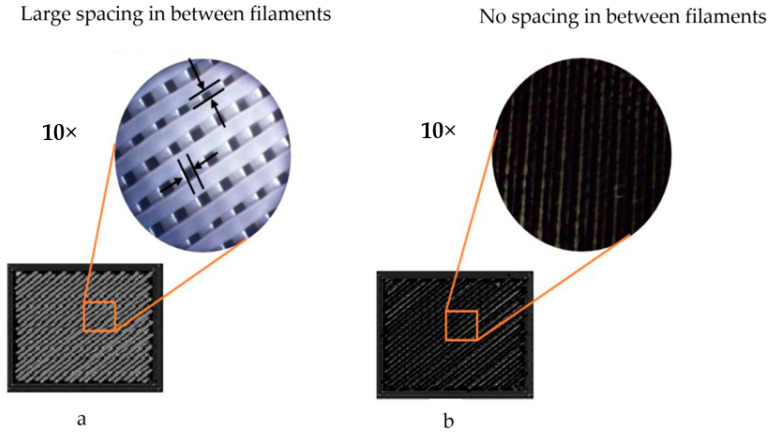
Internal structure of (**a**) density infill 70%, (**b**) density infill 100%.

**Figure 4:**
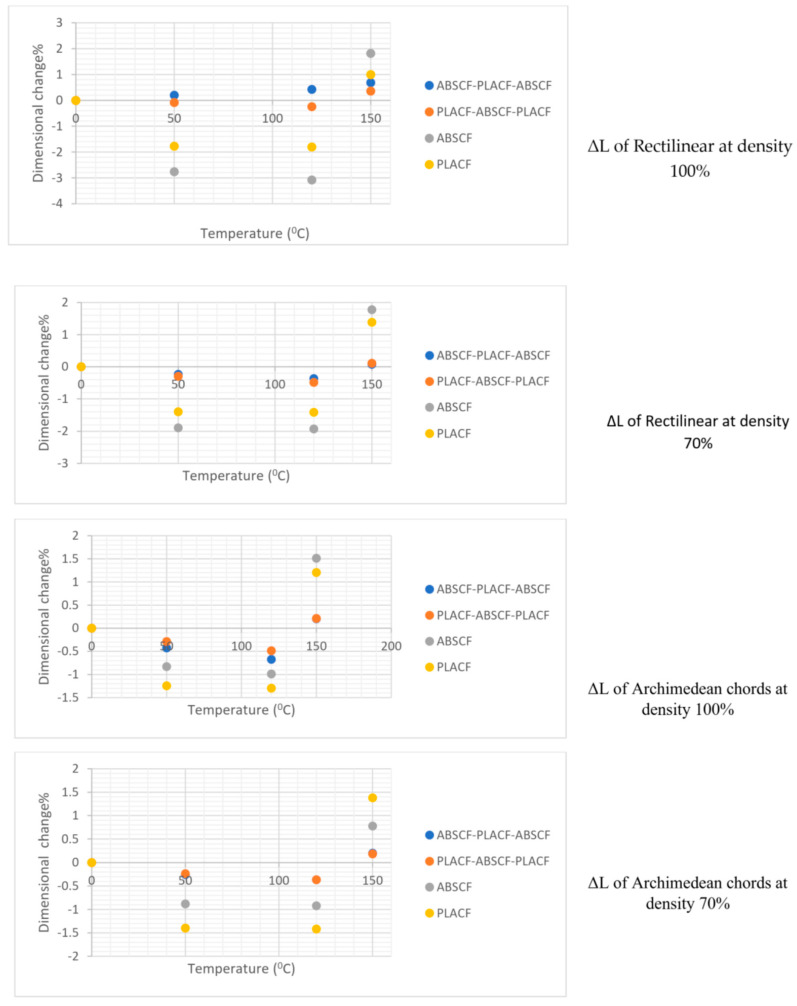
Relative change values of width (L) for the samples under different heat-treatment conditions.

**Figure 5:**
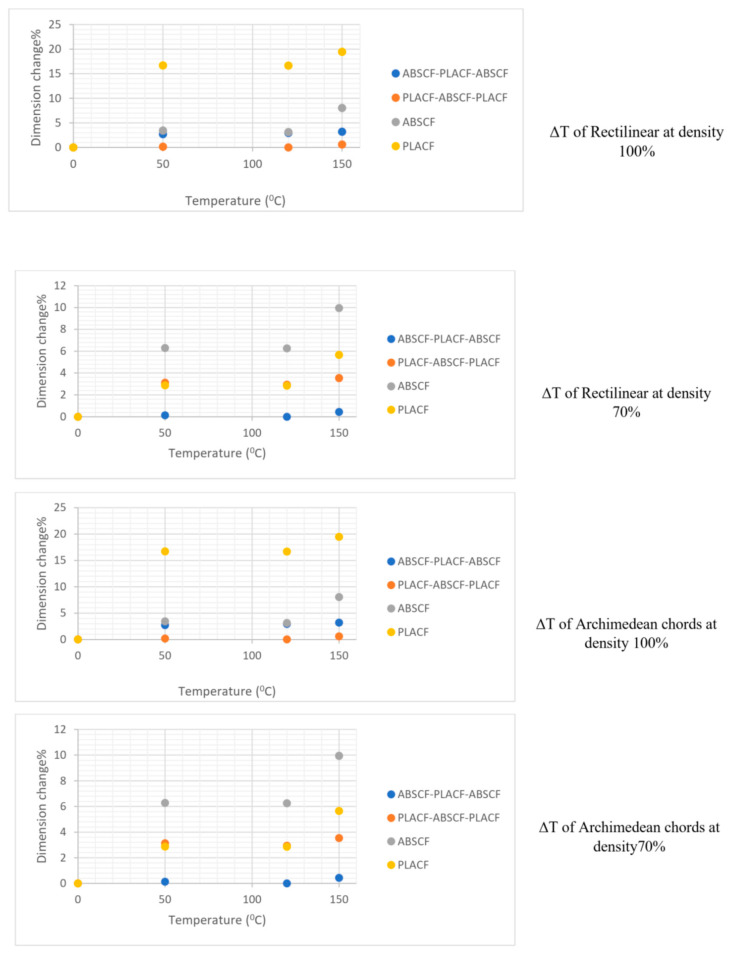
Relative change values of thickness (T) for the samples under different heat-treatment conditions.

Figure 3 shows the internal structure of the sample with density infill 70% and 100%, the large distance between filaments in the case of 70% infill density and the proximity between the filaments in case of infill 100% is clear from the figures. Figure 4 and Figure 5 show relative change values of width (L) and thickness (T) for the composites under different heat-treatment conditions, respectively. It was found that increasing the temperature of heat treatment up to 150 °C leads to a decrease in the width and thickness in all samples. For example, the percentage dimensional change of ABSCF-PLACF-ABSCF/rectilinear pattern/100 was 0.19% at 50 °C and 0.68 at 150 °C. In addition to that, the density of infill was an important parameter in the % of relative change value of the sample width and thickness. With the decrease of infill density up to 70%, the width and thickness of the sample tend to decrease. The maximum change in dimension was 19.46% found in PLACF/rectilinear/100 with thickness after 150 °C heat-treatment. The results also revealed that the pattern shape was an important parameter in the % of relative change value of the sample width and thickness. Samples with Archimedean chords pattern shape showed lower % of relative change value of the sample width and thickness compared with samples with rectilinear pattern. For example, the % dimensional change of ABSCF/Archimedean chords/100 was 11.07% while the % dimensional change of the rectilinear pattern was 8.02 at the same heat treatment temperature.

### 3.2. Mechanical Properties

#### 3.2.1. Tensile Strength

**Figure A4:**
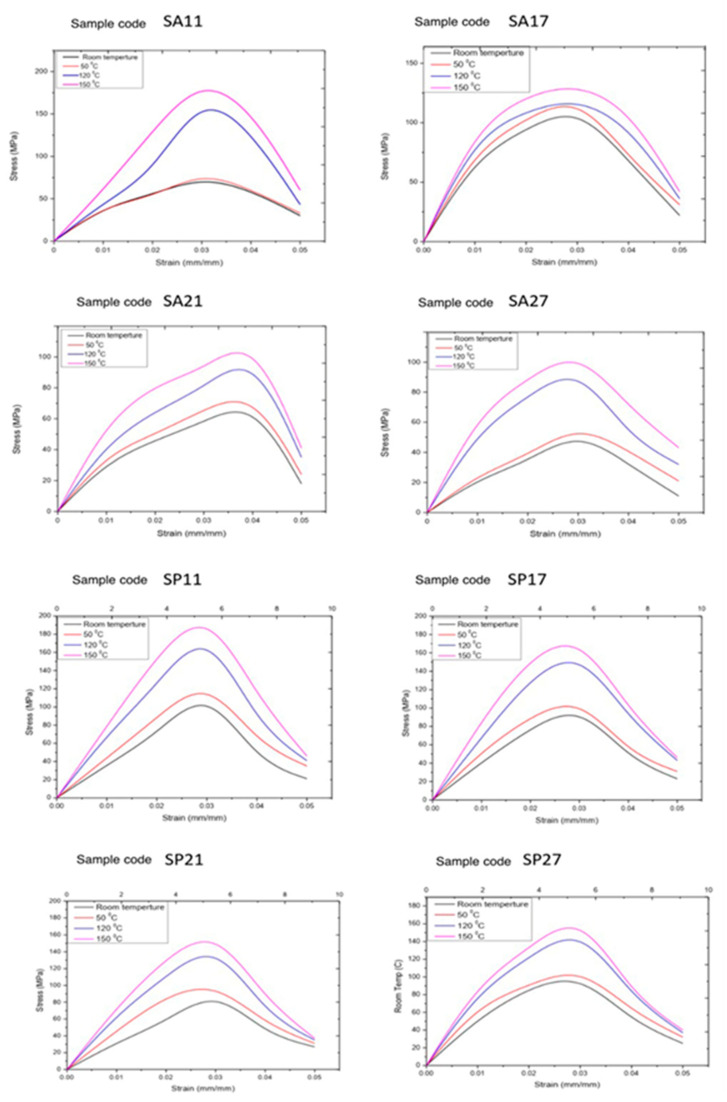
Tensile strength testing results of type A samples.

**Figure A5:**
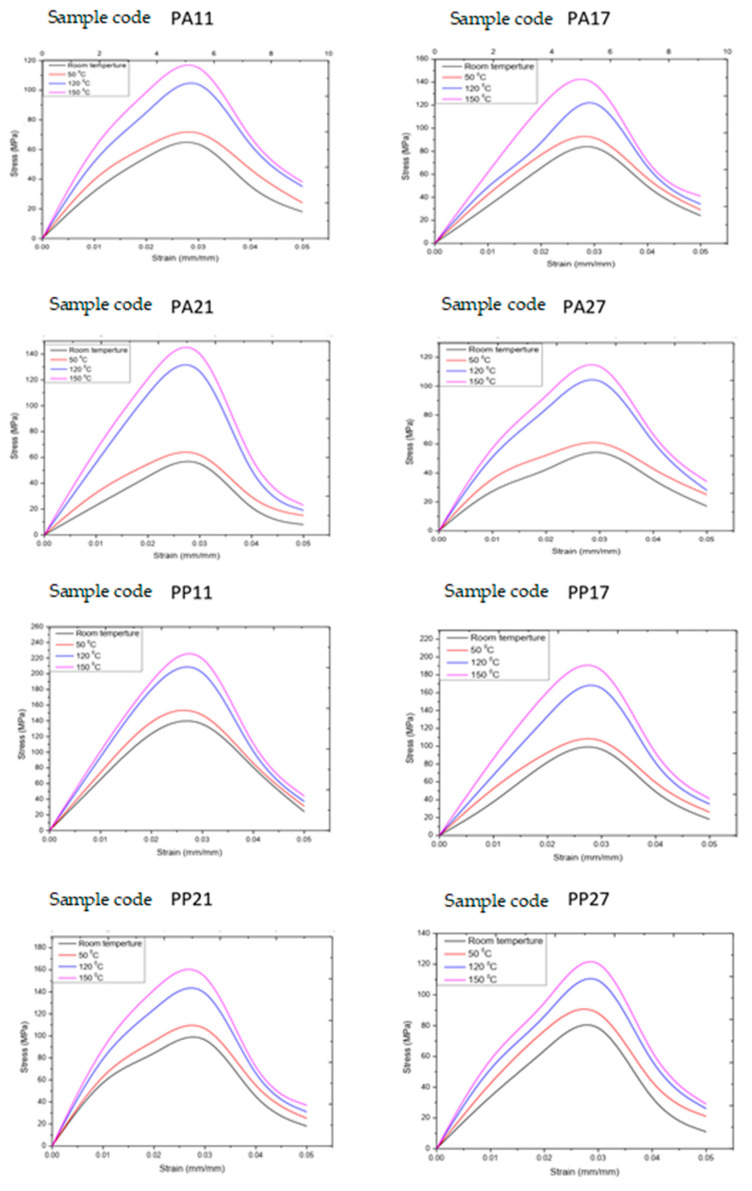
Tensile strength testing results of type B samples.

Figure A4 and Figure A5 represent the results of tensile strength testing for samples of types A and B. It is clear from the figures that heat-treated samples had a remarkable increase in stiffness and strength compared with the non-treated samples. The maximum tensile strength for heat treatment samples was 257.4 MPa for sample PP11 at 150 °C and the minimum tensile strength was 15.02 MPa obtained for sample PA21 treated at 50 °C. Heat treatment at 150 °C shows a remarkable increase in the tensile strength; at this temperature, samples exhibited 155.24% increased strength over the untreated samples in the case of ABSCF-PLACF-ABSCF with 100% rectilinear infill. This was due to a delay in the crack initiation for the heat-treated samples under the effect of decreasing porosity at filament layers, which led to increasing the sample strength [3]. In addition to enhancement of tensile strength, samples treated at 150 °C show an increase in Young’s modulus of elasticity; at this temperature, the samples displayed 12.5% increase in Young’s modulus of elasticity over the untreated samples in the case of PLACF-ABSCF-PLACF with 100% Archimedean chords infill. The results also reveal that there is a significant correlation between the heat treatment temperature and tensile properties. This is due to the limited improvement in the tensile strength and modulus of elasticity values for the samples treated at low heat-treatment temperature compared with samples treated at high temperatures. On the other hand, the maximum tensile strength for untreated samples was 151.7 MPa at sample PP21 and the minimum tensile strength was 54.39 MPa obtained in the SA27 sample.

#### 3.2.2. Porosity Measurement

**Figure 6:**
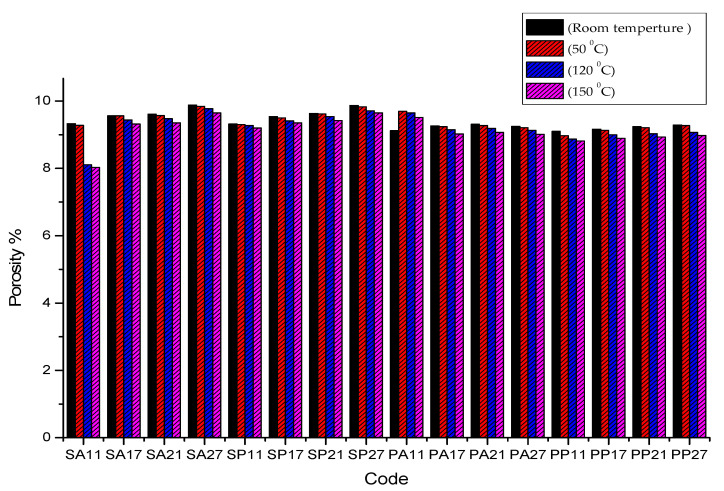
variation of the porosity % with the changing in the heat treatment temperature.

**Figure 7:**
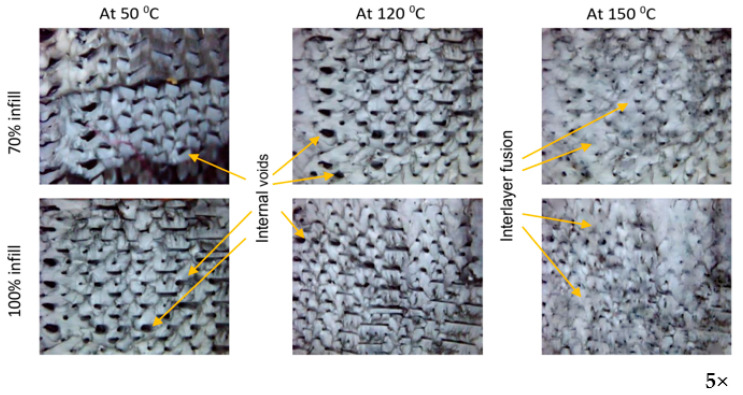
Microscopic images of the cross-section of the samples at different temperatures for heat treatment.

Figure 6 shows the effect of heat treatment on the porosity % of different samples, it illustrates the values of porosity % at different heat treatment temperatures. It is clear from the bars values that the porosity decreases with the increase of heat treatment temperature. For example, the porosity % of sample SA11 was 9.28% at 50 °C and 8.03 at 150 °C. In addition to that, the density of infill was an important parameter in the porosity %. With the decrease of infill density up to 70%, the porosity % tended to increase. Furthermore, the pattern of the filament has a significant effect on the porosity %; rectilinear pattern shows lower porosity % compared with the Archimedean chords pattern. For example, the porosity % of sample PA27 was 9.12% and 9.31% for PA21. In addition, samples with the same filament type show lower porosity % compared with the samples with different filament types. The minimum value of porosity % was 8.03% for sample SA11 treated at 150 °C and the maximum value of porosity % was 9.88% obtained in untreated sample SA27. The previous results agree with the microscopic images of the cross-section of the samples at the different temperatures for heat treatment in Figure 7. Microscopic image analysis reveals that internal voids were reduced with the further decrease in heat treatment temperature. In other words, when the sample is treated at a temperature near to the glass transition of polymer, the motion of the molecule becomes sufficient to fill voids and to enhance interlayer fusion.

### 3.3. SEM Analysis

In order to study the effect of heat-treatment on the failure response of samples, scanning electron microscope (SEM) was used to fracture surfaces of tensile test samples. Six samples were chosen according to the best and worst tensile behavior given by them. These samples are PP11 as the best ultimate strength for unannealed specimens andPP11-A the best strength for annealed specimens. The highest elasticity specimens which are SA21 for unannealed specimens and SA11-A for annealed specimens. The worst strength is the SA27 and the lowest elasticity is SP17-A. The scanning was performed twice for each specimen at magnifications of 50 and 200. 

**Figure 8:**
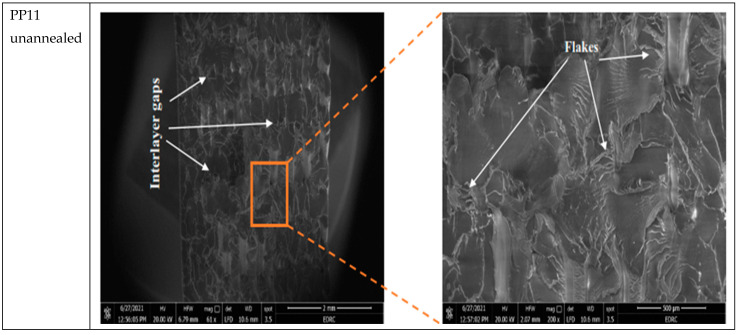

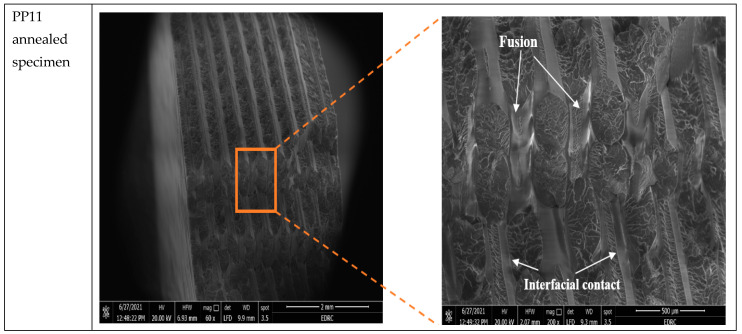
SEM photos and EDS plot of the effect of heat treatment over PP11 sample.

Figure 8 shows the SEM images for the PP11 specimen (PLACF with 100% rectilinear infill) which gives the highest ultimate strength among all the other unannealed specimens. Here the pure rectilinear PLACF shows flake structure at the outer layers and complete merger between filaments due to complete fusion in the filament interlayer; while the overall crosslinking is strong which gives high ultimate strength value. On the other hand, annealed PP11 gives the highest ultimate strength among all the other annealed specimens with ultimate strength equal to 46 MPa. By comparing those specimens, it is noticeable that the filament material becomes more fusional in the annealed samples than unannealed samples, which makes excellent interfacial contact between layers. In other words, heat treatment reheats the filament which improves the crosslinking between filament surfaces compared with the crosslinking of filament in the same sample without an annealing process. 

**Figure 9:**
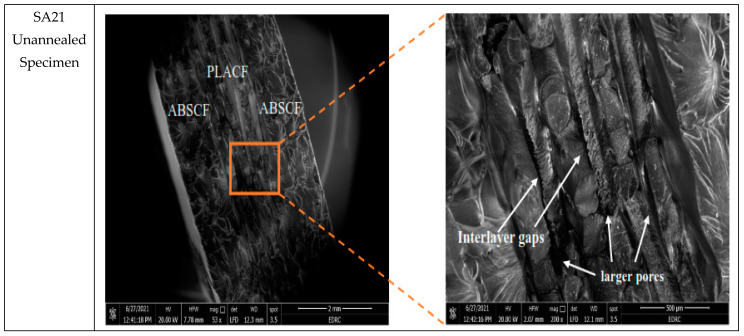

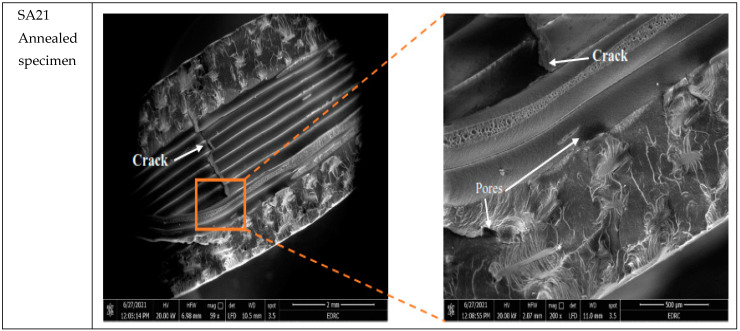
SEM photos for the effect of heat treatment over SA21sample.

**Figure 10:**
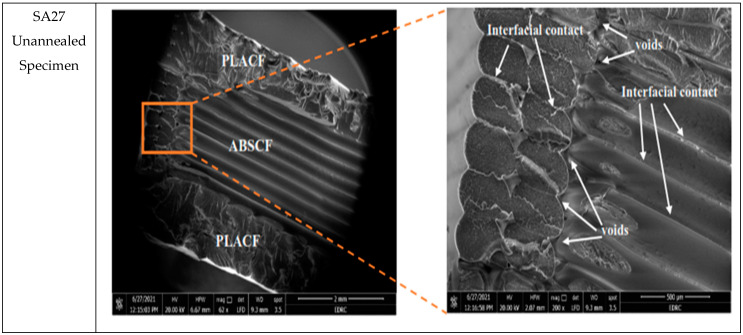
SEM photos of unannealed SA27specimen.

**Figure 11:**
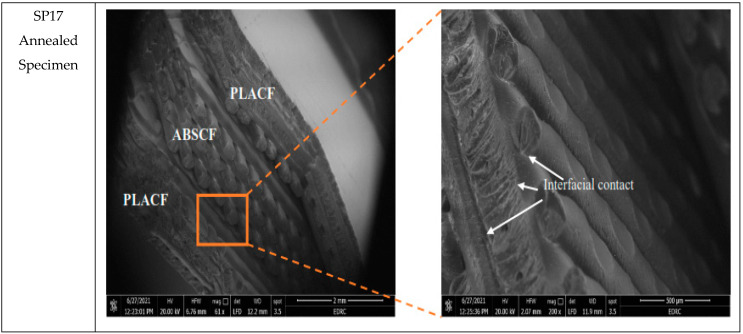
SEM photos for the effect of heat treatment over SP17sample.

Figure 9 shows the SEM of the SA21 (PLACF-ABSCF-PLACF with 100% Archimedean chords infill) which gives the highest Young’s modulus among all the other unannealed specimens (1.49 GPa). The material here is ductile as seen in the fracture surface, this is due to appearance of the transition region with hackles and mists [42]. Meanwhile, the surface of fracture for the heat treatment sample SA11 shows shiny and smooth surface which refers to fusion or remelting of the outer surface of the filament material. Furthermore, the surface of the fracture failure after heat treatment was brittle due to the plastic deformation in the filament material under the temperature of heat treatment. Figure 10 shows the SEM of the SA27 (ABSCF-PLACF-ABSCF with 70% Archimedean chords infill) which gives the lowest ultimate strength among all the other annealed and unannealed specimens. Here the sandwich specimen, which has an ABSCF outer layer, exhibits the lowest tensile strength as it has a lot of voids and poor crosslinking between filament surfaces. The SEM images reveal the appearance of voids in the filament layer of ABSCF and filament layer of PLACF due to fusion temperature deference between them. However, interfacial contact appears between the same types of filaments, due to same thermal properties. Figure 11 shows the SEM photos of SP17 (PLACF-ABSCF-PLACF with 70% rectilinear infill) which gives the lowest Young’s modulus among all the other annealed and unannealed specimens. This behavior is due to the bad bonding between dissimilar materials of the filaments. It is clear from the SEM image that the interfacial contact or the adhesion appears only between the same type of filament. On the other hand, bad tensile properties of the sandwich from different types of filaments have resulted from the mismatched strain between the layers. 

## 4. General Comments

This paper analyses the effect of heat treatment at three different temperatures on the physical and tensile behavior of two types of filaments printed by using two infill densities and two patterns infill. The percentages of porosities shown correlated with densities and patterns of infill; this is due to differences in thermal expansion of the filament which affects interfacial bonding between filament layers, the other reason for the porosity is the lack in filling to complete the geometric space between filaments in case of the small density (70%) [43]. It is clear from Figure 3 that there is a large space between the filaments in the case of density infill 70%, while the filaments are very close to each other in the case of density infill 100%.

The rectilinear pattern shows lower porosity compared with the Archimedean chords pattern as a result of the design of its structure which consists of square prismatic channels separated by perpendicular walls and parallel walls. This structure provides low spacing between filament interfacials which reduces the possibility of pores creation, the same observation was found by Keles [44]. The most critical porosity due to intra bead pores can be eliminated by enhancing the adhesion between fiber and matrix and using high-quality filaments [45].

**Figure 12:**
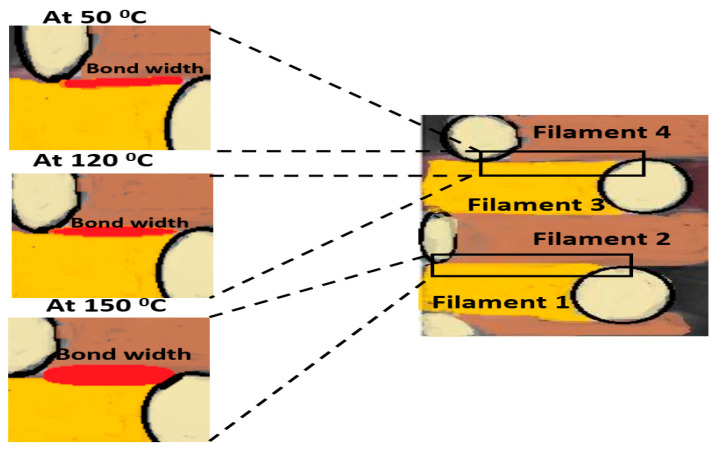
Effect of heat treatment temperature on the contacting surface of the filaments.

Reduction in the tendency of porosity for printed samples after the heat treatment process is returned to shrinkage and creeping in the polymer interfacial [46,47,48]. Although selecting a suitable heat treatment temperature is essential in the improvement of mechanical properties [49], if the heat treatment temperature is less than the melting temperature of the filament, the porosity will remain as it is without any changes. Thus, the mechanical properties will not improve. Moreover, the main affected parameter with the heat treatment is the crystallization temperature of the filament, therefore the changes in porosity will occur only if there is a change in filament crystallinity after the heat-treatment process [50,51]. This increase in the tensile strength and the modulus of elasticity for the samples treated at high temperatures is due to the improvement in the physical touching between layers. In other words, the high temperature helps in remelting the outer surface of the filament (Figure 12), and as a result, the contact surfaces between filaments become more adhesive with each other. It is clear from the schematic that the contacting surface or the width of bonding of the filaments remelts during the heat treatment process. However, the complete merge between filaments appeared at 150 °C due to complete fusion in the filament interlayer. Another possible explanation for the increased tensile strength is that the crack must transfer along the sample width as well as perpendicular to filament layers. In other words, the amount of impact resistance of 3D-printed polymer composite depends on properties of the material and the interfacial bonding between each layer [36].

**Figure 13:**
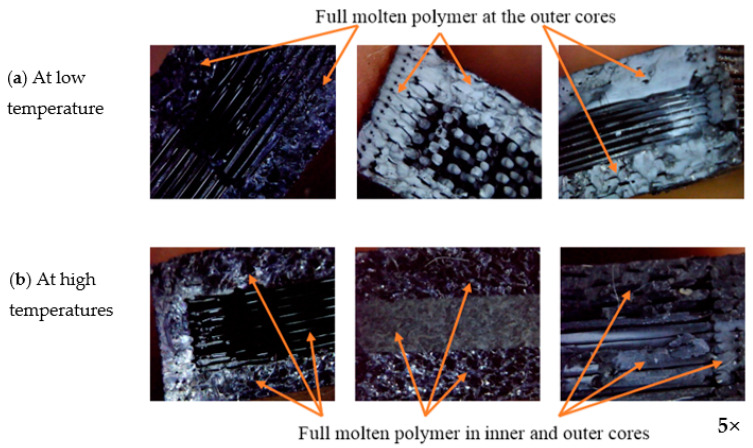
Effect of heat treatment on the surfaces of different samples, (**a**) At low temperature (50 °C), (**b**) At high temperatures (120, 150 °C).

In that case, the fracture area will increase which leads to an increase in the sample toughening, and thus the crack took a long time to travel. The combination between two types of filaments shows excellent tensile properties in the case of using two layers of PLACF in the upper and lower layers, and using one layer of ABSCF in the middle of the sample. This is due to PLACF having higher strength than ABSCF. The differences in obtained tensile strength with the changes in heat treatment temperatures may be due to the following reasons:

1. At low temperature (50 °C), only the outer surfaces of the samples were affected by heat treatment; thus, the melting material appeared there (Figure 13a),
2. At high temperatures (120 and 150 °C), the polymer reaches its melting point temperature; thus, the melting material appeared all over the sample (Figure 13b).

**Figure 14:**
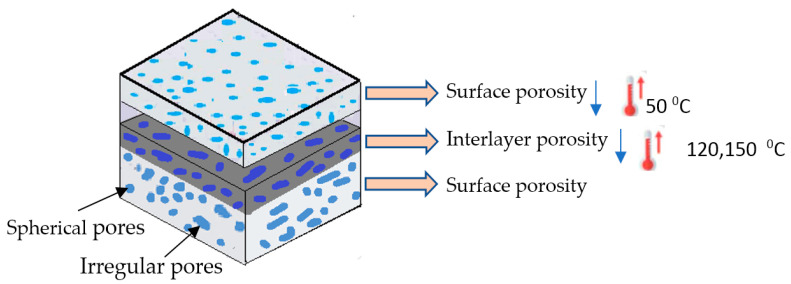
Effect of heat treatment temperature on types and locations of pores.

The mechanical performance of 3D-printed products depends on the quality of microstructural morphology which is affected by the porosity of the content and distribution of air pores [52]. The interlayer pores are critical to the tensile properties of the 3D-printed sample; if it is subjected to tensile force across the layer containing those inter pores, the sample will break easily [53]. This is due to inter pores acting as defects inside the product under tensile loading [53,54,55,56]. Heat treatment is one of the suitable techniques to eliminate this defect; the air voids will fill with molten material under high temperatures during heat treatment (Figure 14). In the current study, three temperatures were used: low, medium, and high. Low temperature succeeds in removing only some of the surface defects, however medium and high temperatures helped in curing internal defects by reducing the random spherical pores and improving the mechanical properties.

## 5. Conclusions

In this work, the effects of the heat-treatment process on the physical and mechanical properties of 3D-printed samples using different types of filaments and with different pattern shapes and densities have been examined. Based on the experiments, the following results could be mentioned

- Among the different combinations of input parameters considered for the study, the combination between two types of filaments shows excellent tensile properties in the case of using PLACF as upper and lower layers, and using one layer of ABSCF in the middle,
- Decreasing density of infill up to 70% leads to increasing increase in the porosity content,
- The best heat-treatment condition for PLACF is 120 °C and for ABSCF is 150 °C. Under those temperatures, the PLACF and ABSCF filament show maximum tensile strength and low porosity content,
- The maximum tensile strength for heat treatment (at 150 °C) samples was 257.4 MPa at sample PLACF/rectilinear pattern and 100% density,
- There is limited improvement in the tensile strength and modulus of elasticity values for the samples treated at low heat-treatment temperature compared with samples treated at high temperatures,
- At all structure specifications, the rectilinear pattern gives higher strength of up to 33%. This is due to the linear shape of the rectilinear pattern being better than the concentric circular shape of the Archimedean chords pattern,
- Untreated and Archimedean chords pattern exhibited higher porosity % compared with untreated rectilinear pattern. However, in the case of the heat-treated samples, more interfacial contact and fusion occurs.

## Figures and Tables

**Table 1:** Properties of polymer ABSCF and PLACF.

Filament Type	Density g/cc	Tensile Strength (MPa)	Tensile Modulus (MPa)	Tensile Elongation (%)	Flexural Strength (MPa)	Extrusion Temp	Glass Transition Temperature
PLACF	1.29	48	4950	2	89	215 °C	60 °C
ABSCF	1.11	46	5210	2	76	230 °C	105 °C

**Table 2:** Printing parameters.

Infill Pattern	Density %	Feed Rate (E/mm min^−1^)	Printing Speed (E/mm min^−1^)	Bed Temp. °C
Rectilinear	100/70	80	100	PLACF: 23 °C
Archimedean chords	100/70	80	100	ABSCF: 110 °C

**Table 3:** Sample codes.

Material	Infill Pattern	Density	Code
ABSCF-PLACF-ABSCF	Rectilinear	100	SA11
ABSCF-PLACF-ABSCF	Rectilinear	70	SA17
ABSCF-PLACF-ABSCF	Archimedean chords	100	SA21
ABSCF-PLACF-ABSCF	Archimedean chords	70	SA27
PLACF-ABSCF-PLACF	Rectilinear	100	SP11
PLACF-ABSCF-PLACF	Rectilinear	70	SP17
PLACF-ABSCF-PLACF	Archimedean chords	100	SP21
PLACF-ABSCF-PLACF	Archimedean chords	70	SP27
ABSCF	Rectilinear	100	PA11
ABSCF	Rectilinear	70	PA17
ABSCF	Archimedean chords	100	PA21
ABSCF	Archimedean chords	70	PA27
PLACF	Rectilinear	100	PP11
PLACF	Rectilinear	70	PP17
PLACF	Archimedean chords	100	PP21
PLACF	Archimedean chords	70	PP27
